# The 3D Pattern of the Rainbow Trout (*Oncorhynchus mykiss*) Enterocytes and Intestinal Stem Cells

**DOI:** 10.3390/ijms21239192

**Published:** 2020-12-02

**Authors:** Nicole Verdile, Rolando Pasquariello, Tiziana A. L. Brevini, Fulvio Gandolfi

**Affiliations:** 1 Department of Agricultural and Environmental Sciences, University of Milan, 20133 Milano, Italy; Nicole.verdile@unimi.it (N.V.); Rolando.pasquariello@unimi.it (R.P.); 2 Department of Health, Animal Science and Food Safety, University of Milan, 20133 Milano, Italy; tiziana.brevini@unimi.it

**Keywords:** rainbow trout, intestinal epithelium, intestinal stem cells, self-renewal, fish nutrition, brush border proteins

## Abstract

We previously showed that, according to the frequency and distribution of specific cell types, the rainbow trout (RT) intestinal mucosa can be divided in two regions that form a complex nonlinear three-dimensional (3D) pattern and have a different renewal rate. This work had two aims. First, we investigated whether the unusual distribution of cell populations reflects a similar distribution of functional activities. To this end, we determined the protein expression pattern of three well-defined enterocytes functional markers: peptide transporter 1 (PepT1), sodium–glucose/galactose transporter 1 (SGLT-1), and fatty-acid-binding protein 2 (Fabp2). Second, we characterized the structure of RT intestinal stem-cell (ISC) niche and determined whether the different proliferative is accompanied by a different organization and/or extension of the stem-cell population. We studied the expression and localization of well-characterized mammal ISC markers: LGR5, HOPX, SOX9, NOTCH1, DLL1, and WNT3A. Our results indicate that morphological similarity is associated with similar function only between the first portion of the mid-intestine and the apical part of the complex folds in the second portion. Mammal ISC markers are all expressed in RT, but their localization is completely different, suggesting also substantial functional differences. Lastly, higher renewal rates are supported by a more abundant ISC population.

## 1. Introduction

Due to its high nutritional value, global fish consumption has constantly grown in the past few decades, reaching all-time records. Whereas the production of capture fisheries has reached its plateau, aquaculture output has increased fivefold in the last 30 years, becoming the biggest source of the fish we eat [1]. Nevertheless, intensive fish farming must constantly evolve in order to increase its sustainability, particularly through the search for feed with low environmental impact. However, many of the alternative and sustainable ingredients have a negative impact on the integrity and function of fish intestine [2,3]. Therefore, it is important to increase our knowledge on its structure and on the mechanisms that regulate the mucosa renewal and repair.

Fish represent the largest group of vertebrates, including almost 21,000 species, more than all other vertebrates combined; they are characterized by a wide variety of anatomical and physiological differences [4]. We recently examined in detail the intestinal structure of rainbow trout (RT; *Oncorhynchus mykiss*) [5], a member of the Salmonidae family, one of the most successful groups in aquaculture by virtue of its adaptability to a wide range of farming conditions [6]. Following the nomenclature proposed by Bjørgen et al. [7] for the Atlantic salmon (*Salmo salar*), along the craniocaudal axis of the intestine, we first meet the pyloric caeca, over 50 hollow tubes that branch off the first segment of the mid-intestine, to increase its absorptive surface. These are followed by the rest of the thin and nearly transparent first segment of the mid-intestine that continues in the second segment of the mid-intestine, easily recognizable for its larger diameter and darker pigmentation due to the presence of circularly arranged blood vessels. Fish intestinal mucosa lacks two typical structures found in mammals: the crypts of Lieberkühn, and the villi [8]. Throughout the length of the whole intestine, the mucosa raises in large folds, and, in the second segment, some of them are up to three times higher with smaller secondary folds branching out along their length. For their peculiar structure, these are known as complex folds [5,9]. 

We previously described a peculiar distribution pattern of the different cell populations along rainbow trout intestinal mucosa [5]. The epithelium covering the apical part of the complex folds of the second segment presented abundant and actively secreting goblet cells and no pinocytotic vacuoles in the first segment of the mid-intestine, whereas the pyloric caeca and the basal part of the complex folds in the rest of second segment of the mid-intestine were lined with an epithelium characterized by few deflated goblet cells and high pinocytotic vacuolization. Moreover, these two intersected districts showed distinct turnover rates: the first segment of the mid-intestine and the apical part of the complex folds were characterized by low proliferation and extensive differentiation, while the contrary occurred in the pyloric caeca, the basal part of the complex folds, and the rest of the second segment of the mid-intestine [5]. 

The self-renewal capability of the intestinal epithelium is ensured by a dedicated population of multipotent intestinal stem cells (ISCs), which in mammals are in the well-demarcated space of the Lieberkühn crypts that form a highly specialized supporting niche [10,11,12,13]. To date, by far the most detailed knowledge on ICSs and their interaction with the surrounding niche has been achieved in mouse [12,14,15]. Two different stem-cell populations coexist in this species: the leucine-rich-repeat-containing G-protein-coupled receptor 5-expressing (LGR5^+^) stem cells and the +4-stem cells so called because are located in the fourth position from the bottom of the crypt that selectively express homeobox only protein (HOPX) [16]. LGR5^+^ cells, also called crypt base columnar cells (CBCs), are continuously cycling cells located at the crypt base intermingled among Paneth cells [13,15,17,18]. HOPX^+^ cells are located in the fourth position from the bottom of the crypt, characterized by label retaining and slow cycling [7]. They enter in action in response to a damage of the epithelium, and most recent data suggest that they can integrate the CBC population. ISC cell division originates a pool of highly proliferating cells known as transient amplifying (TA) cells that expand rapidly to produce terminally differentiated cells that migrate up toward the villus tip with the only exception of Paneth cells that migrate toward the crypt. On the LGR5^+^ cell surface, the NOTCH 1 receptor is also present, which interacts with its transmembrane ligand Delta-like protein 1 (DLL1) located on Paneth cells. The two together play a crucial role in regulating stem-cell proliferation and differentiation [19]. ISC maintenance is also regulated by other pathways, such as WNT3A, which is a ligand of LGR5 and acts together with the Notch pathway to modulate stem-cell equilibrium between proliferation and differentiation [15].

Whereas detailed knowledge of ISCs and their crypts is growing exponentially in mouse and in humans, very little is known in other species [11,20]. Among fish, the organization and the architecture of the intestinal stem-cell niche were recently investigated only in medaka [8] and in zebrafish [21,22,23]. However, these are two small laboratory species and, as mentioned above, the taxonomic divergence among teleost fish does not allow direct extrapolations.

Given the complex nonlinear three-dimensional (3D) pattern of the RT intestine that is not observed in mammals, this manuscript had two aims. The first was to investigate whether the unusual distribution of cell populations reflects similar functional activities. To this end, we determined the protein expression along the different portions of the intestine of three well-defined functional markers of differentiated enterocytes: peptide transporter 1 (PepT1), sodium–glucose/galactose transporter 1 (SGLT-1), and fatty-acid-binding protein 2 (Fabp2). The second was to characterize the morphological and molecular structure of the RT intestinal stem-cell niche and to determine whether the different proliferative rates previously observed along the different mid-intestine portions correlates with a different organization and/or extension of the stem-cell population.

## 2. Results

### 2.1. Distribution of Functional Brush Borders Proteins along the Mid-Intestine

**Figure 1:**
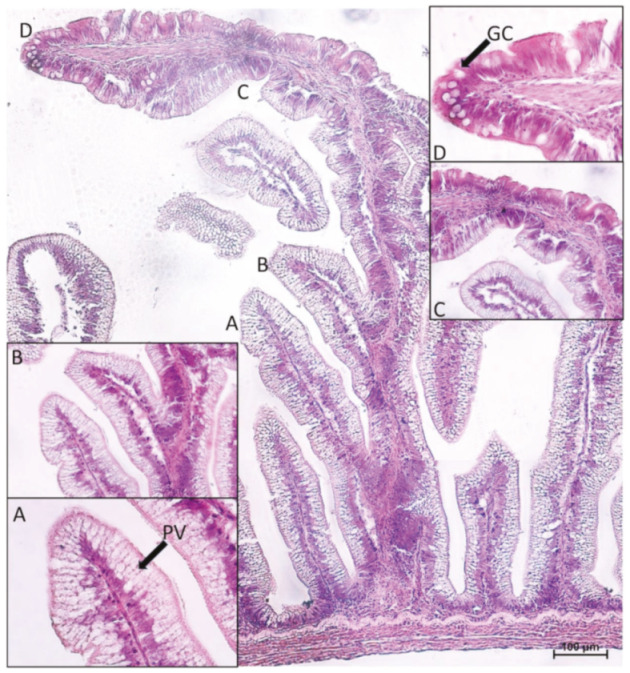
Morphological aspects of the complex folds found in the second segment of the rainbow trout mid-intestine. Basal part (**A**,**B**), characterized by a great amount of pinocytotic vacuolization (PV) that gradually disappeared (**C**). Apical portion (**D**), characterized by numerous goblet cells (GC) and no vacuolization. Hematoxylin–eosin paraffin section.

A preliminary morphological analysis did not evidence morphological alterations such as proximal intestine vacuolization, nuclear positioning disparity, fold shortening, or fold branching, confirming the animals’ healthy state. It confirmed that goblet cells were numerous, swollen, and actively secreting in the first segment of the mid-intestine and in the apical portion of the complex folds of the second segment, whereas they were scarce and inactive in the pyloric caeca and in the rest of the second segment of the mid-intestine. As previously observed, enterocytes full of pinocytotic vacuoles were the predominant cell type along the basal portion of the complex folds and in the rest of the second segment of the mid-intestine (Figure 1). 

**Figure 2:**
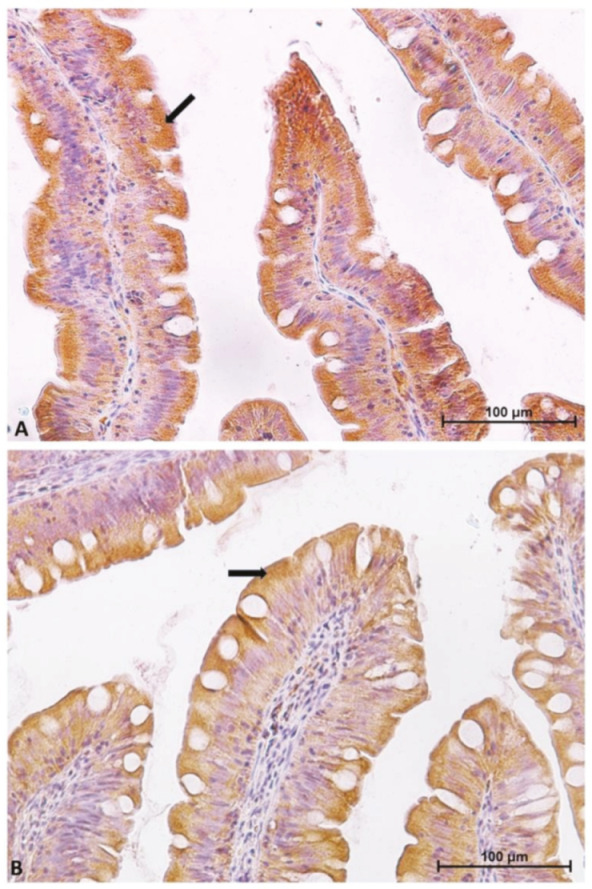
Immunolocalization of peptide transporter 1 (PepT1) in pyloric caeca (**A**) and in the first segment of the mid-intestine (**B**). The molecule is localized in the brush border and in the apical part of the enterocyte cytoplasm (arrows).

**Figure 3:**
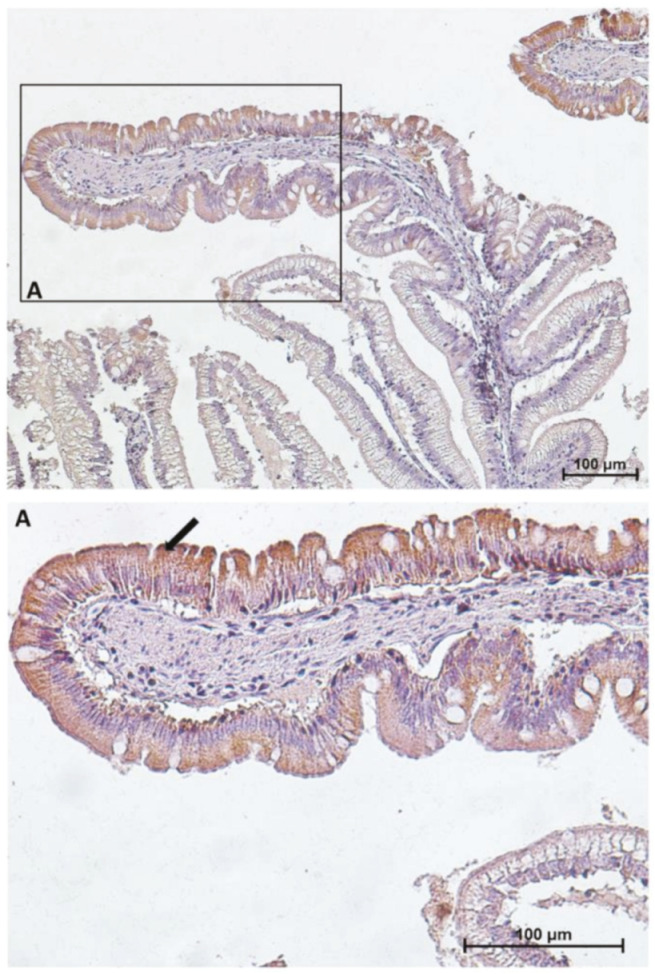
Immunolocalization of peptide transporter 1 (PepT1) in the second segment of the mid-intestine. PepT1 expression is confined to the apical portion of the complex folds where it shows the same intracellular location and signal intensity found in the first segment of the mid-intestine. The lower panel (**A**) shows PepT1 immunolocalization (arrow) at higher magnification.

However, the expression level of peptide transporter 1 (PepT1), sodium–glucose/galactose transporter 1 (SGLT-1), and fatty-acid-binding protein 2 (Fabp2) followed only partially the same pattern. The presence of the PepT1 symporter was highest in the brush border of pyloric caeca enterocytes, was moderate in the first segment of the mid-intestine (Figure 2) and in the apical part of the complex folds of the second segment, and was completely absent in the rest of the second segment of the mid-intestine mucosa (Figure 3). 

**Figure 4:**
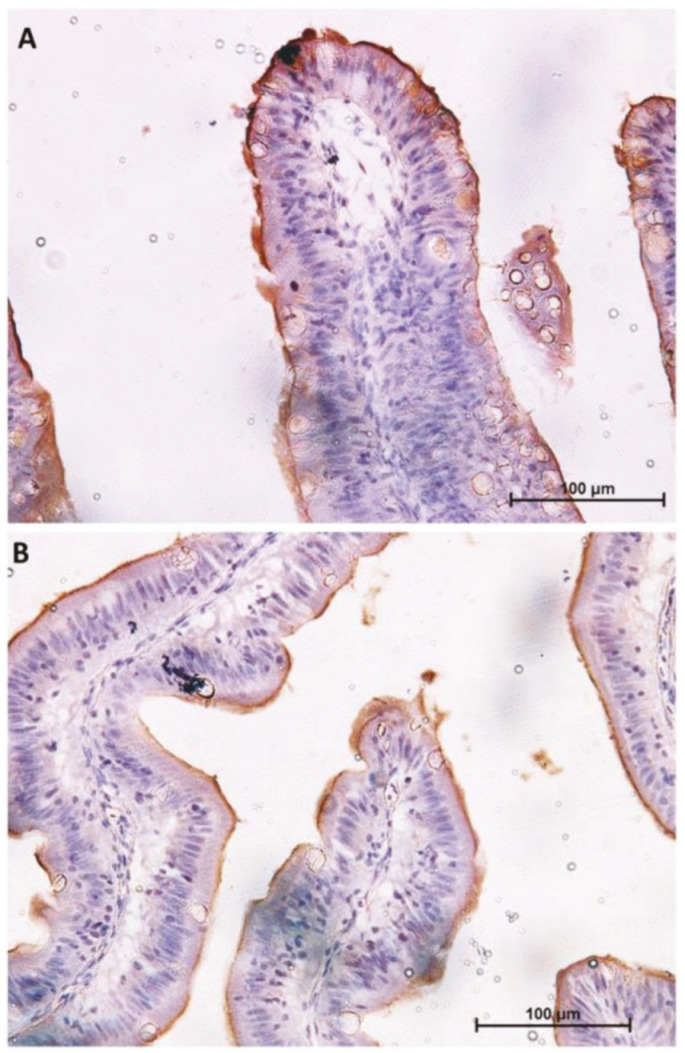
Immunolocalization of sodium–glucose/galactose transporter 1 (SGLT1) in the first segment of the mid-intestine (**A**) and in the pyloric caeca (**B**). The signal is limited to the enterocyte brush border.

**Figure 5:**
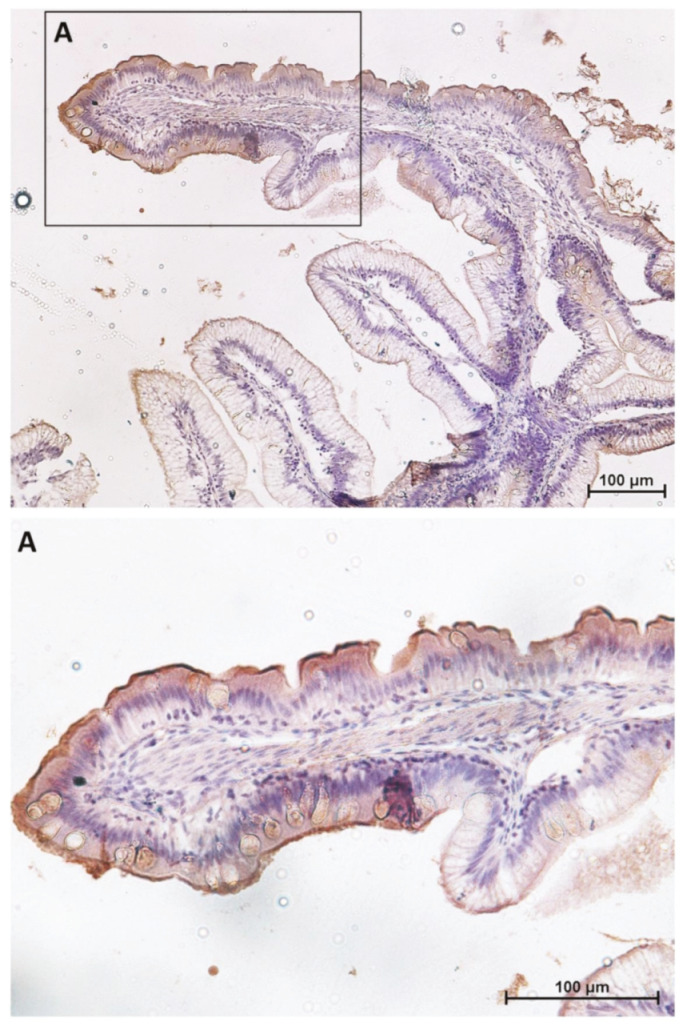
Immunolocalization of sodium–glucose/galactose transporter 1 (SGLT1) in the second segment of the mid-intestine. SGLT1 expression is confined to the apical portion of the complex folds where it shows the same location and signal intensity found in the first segment of the mid-intestine. The lower panel shows (**A**) at higher magnification.

**Figure 6:**
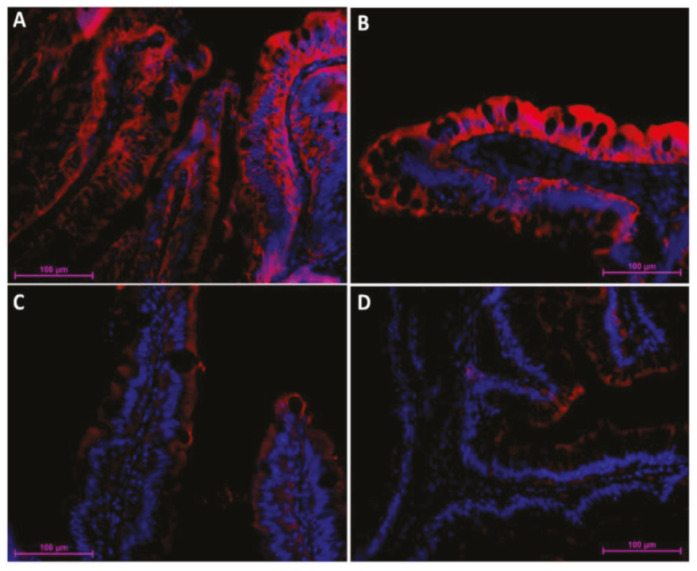
Immunofluorescent localization of fatty-acid-binding protein 2 (Fabp2) along the rainbow trout (RT) intestine (red signal). The signal was most intense in the first segment of the mid-intestine (**A**) and in the apical part of the complex folds (**B**), was intermediate in the pyloric caeca (**C**), and was lowest within the basal part of the complex folds and in the rest of the second segment of the mid-intestine (**D**).

Similar but not identical distribution was observed for SGLT-1 and Fabp2 cotransporter expression. It was highest in the first segment of the mid-intestine and in the apical part of the complex folds of the second segment of the mid-intestine (Figure 4 and Figure 5), was intermediate in the pyloric caeca (Figure 4), and was lowest in the basal part of the complex folds and in the other folds of the second segment of the mid-intestine (Figure 5 and Figure 6).

**Figure 7:**
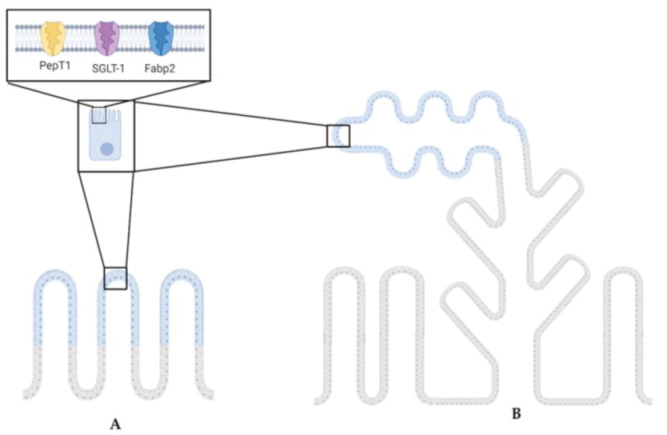
The cartoon highlights the common PepT1, SGLT-1, and Fabp2 expression pattern between the first segment of the mid-intestine (**A**) and the second segment of the mid-intestine (**B**). Illustrations created with BioRender.com.

In summary, cell type distribution and brush border functional protein expression followed the same pattern in the first portion of the mid-intestine and the apical part of the complex folds of the second portion. On the contrary, functional protein expression did not match the cell distribution pattern between pyloric caeca and the rest of the second portion of the distal intestine. Results are schematically summarized in Figure 7.

### 2.2. The Intestinal Stem-Cell Niche Morphological and Molecular Architecture 

We studied the expression of molecules that have been characterized in the mouse intestinal stem-cell niche. LGR5 and HOPX were selected because are well-characterized stem-cell markers. SOX9 is expressed both in stem cells and in partially differentiated cells. NOTCH1 and DLL1 are, respectively, the receptor and the ligand that activate the Notch signaling pathway, which regulates stem-cell maintenance and progenitor cell proliferation. Lastly, WNT3A, a member of the WNT family, induces the formation of the heterodimeric complex of Frizzled and LGR5 that, in turn, activates the expression of genes crucial for stem-cell identity, and its gradient controls the transition from proliferating stem cell to transient amplifying cell toward full differentiation [15,24,25].

#### 2.2.1. Lgr5

**Figure 8:**
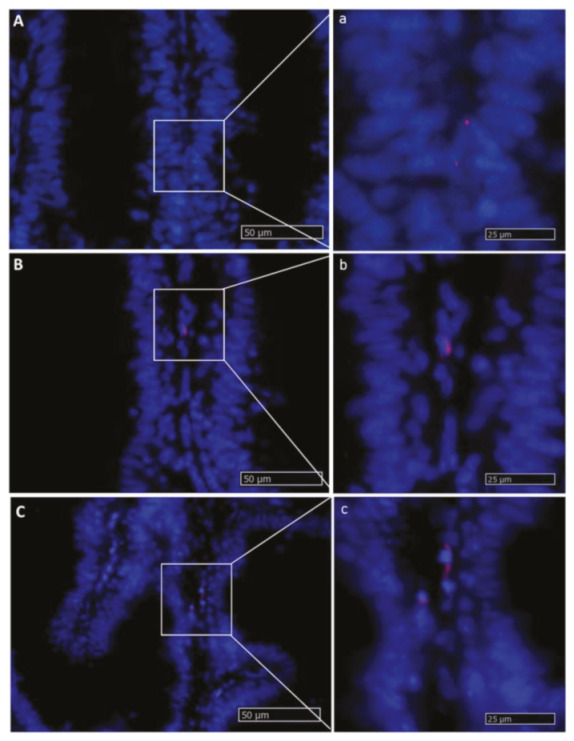
In situ hybridization of *lgr5* messenger RNA (mRNA) in different segments of the rainbow trout mid-intestine (red dots). This gene was expressed at a low rate in the whole organ. The lowest expression level was observed in the first segment of the mid-intestine (**A**,**a**): higher magnification), and an intermediate signal was seen in the pyloric caeca (**B**,**b**): higher magnification), whereas the basal part of the complex folds and the rest of the second segment of the mid-intestine showed the highest signal (**C**,**c**): higher magnification).

*Lgr5*^+^ cells were rare and were never observed in the intestinal epithelium. *Lgr5* was exclusively expressed in scattered cells located in the lamina propria of pyloric caeca, as well as the first and second segment of the mid-intestine. The signal was mostly located at the middle of the fold length and was stronger in the basal part of the complex folds of the second segment of the mid-intestine (Figure 8).

#### 2.2.2. Hopx

**Figure 9:**
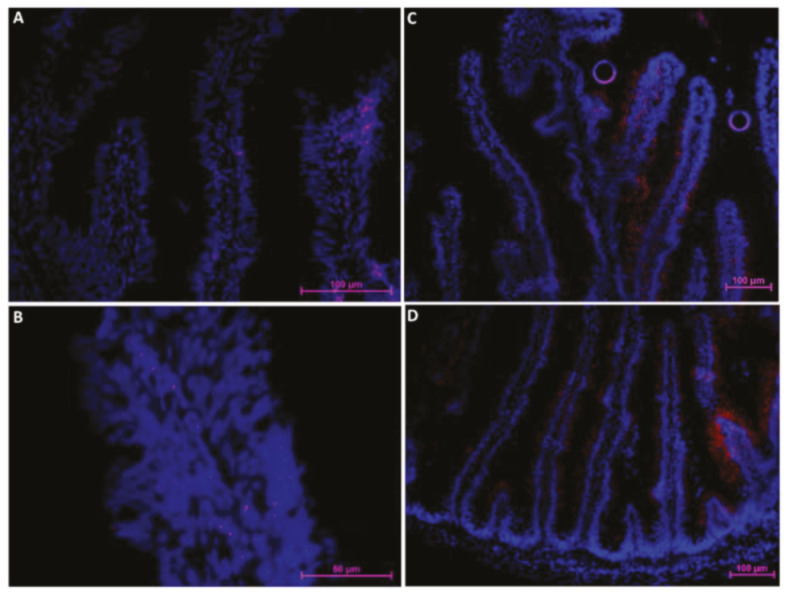
In situ hybridization of *hopx* mRNA in different segments of the rainbow trout mid-intestine (red dots). The signal was moderate in the pyloric caeca (**A**), lower in the first segment of the mid-intestine (**B**,**C**) and in the apical part of the complex folds of the distal, and higher in the basal part of the complex folds (**C**) and the rest of the second segment of the mid-intestine (**D**). The signal tended to disappear at the base and apex of the folds (**A**,**B**).

*Hopx*^+^ cells were detected in the epithelium lining the lower mid-portion of the folds, whereas no signal was detected at the upper portion or at the bottom of the folds. In the other species, this is the usual location of the partially differentiated, transient amplifying population. Moreover, the intensity of *hopx* expression was low in the first segment of the mid-intestine and in the apical part of the complex folds of the second segment of the mid-intestine, while it increased in the pyloric caeca and in the second segment of the mid-intestine, reaching its highest level in the basal part of the complex folds. *hopx* expression was also found in the corresponding lamina propria and as described for *lgr5*, the signal was strongest in the basal part of the complex folds of the second segment of the mid-intestine (Figure 9).

#### 2.2.3. Sox9

**Figure 10:**
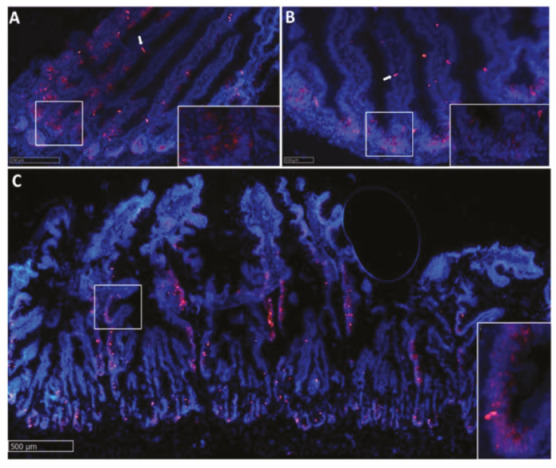
In situ hybridization of *sox9* mRNA cells (red dots) in pyloric caeca (**A**), first segment of the mid-intestine (**B**), and second segment of the mid-intestine (**C**). *Sox9^+^* cells were observed in the epithelium lining the fold base in all the investigated districts.The signal in the pyloric caeca, in the second segment of the mid-intestine, and in the basal part of complex folds was stronger compared to that observed in the first segment of the mid-intestine and in the apical part of complex folds. Isolated *Sox9*^+^ cells were also observed along the fold length (white arrows).

*Sox9* was highly expressed in the epithelial cells lining the fold base along the entire intestine where intestinal stem cells are located in other species. Furthermore, in this case, the signal intensity varied; it was stronger in the pyloric caeca, in the second segment of the mid-intestine, and in the basal part of the complex folds, while it was weaker in the first segment of the mid-intestine and in the apical part of the complex folds (Figure 10). 

**Figure 11:**
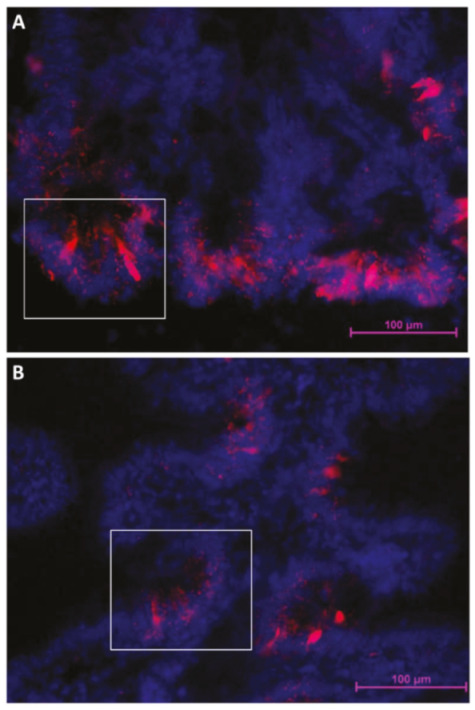
Rainbow trout columnar epithelial cells showing an intense *sox9* expression at the fold base in the second segment of the mid-intestine (**A**) and at the base of the folds protruding from the complex folds (**B**).

Along the whole intestine, at the base of the intestinal folds, we observed slender, epithelial cells expressing *sox9* at very high level (Figure 11). These peculiar-looking cells were homogeneously distributed along the different parts of the intestine. 

**Figure 12:**
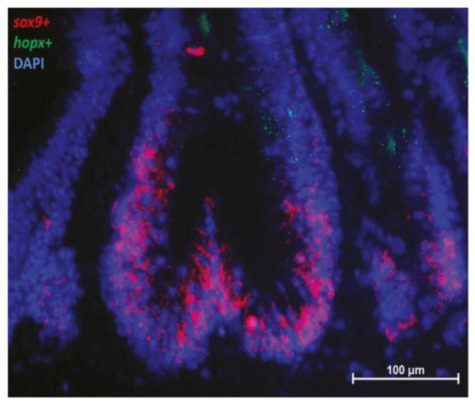
In situ hybridization of *sox9* mRNA (red dots) and *hopx* (green dots) cells in the second segment of the mid-intestine. The two genes were expressed at the base and in the lower half of the fold, respectively.

*Sox9* expression ceased away from the folds base where *hopx* expression was strong, with the two genes never overlapping (Figure 12). Occasionally, however, a few of these cells were also found far from the base along the fold length.

#### 2.2.4. Notch1

**Figure 13:**
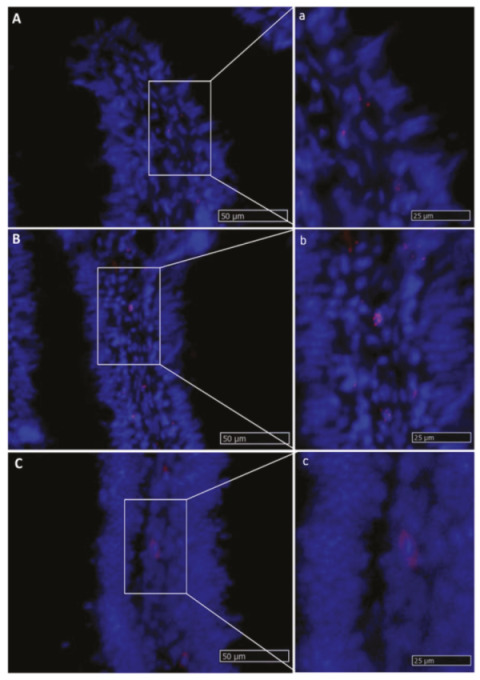
In situ hybridization of *notch1* mRNA expression in different segments of the rainbow trout mid-intestine. The lowest expression was seen in the first segment of the mid-intestine (**A**,**a**): higher magnification), and an intermediate signal was observed in the pyloric caeca (**B**,**b**): higher magnification), whereas the basal part of the complex folds and the rest of the second segment of the mid-intestine displayed the highest signal (**C**,**c**): higher magnification).

**Figure 14:**
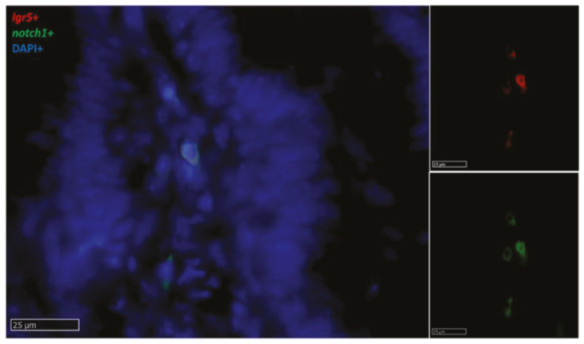
In situ hybridization of *lgr5* (red dots) and *notch1* mRNA (green dots) in the basal part of the complex folds of the second segment of the mid-intestine. Both genes were expressed by a few stromal cells in the fold axis.

In RT, we found *notch1*^+^ cells scattered along the fold connective axis (Figure 13), where it colocalized with *lgr5* in the stromal cells (Figure 14).

#### 2.2.5. Dll1

**Figure 15:**
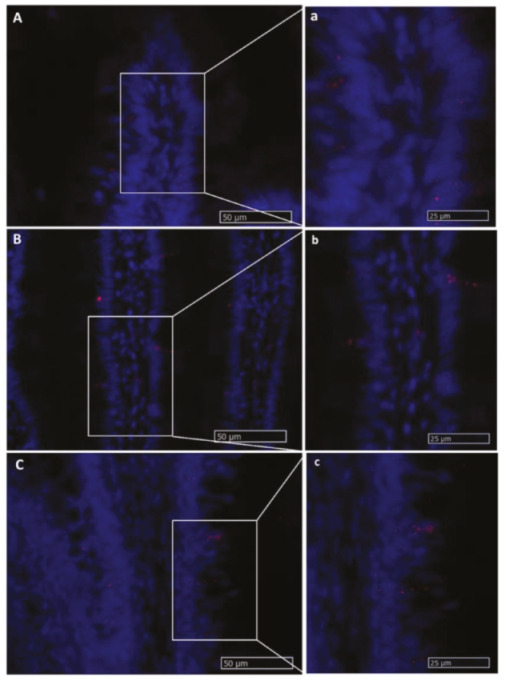
In situ hybridization of *dll1* mRNA in different segments of the rainbow trout mid-intestine. Expression rate was lowest in the first segment of the mid-intestine (**A**,**a**): higher magnification) and intermediate in the pyloric caeca (**B**,**b**): higher magnification), whereas the signal with the highest intensity was found in the basal part of the basal part of the complex folds and in the rest of the second segment of the mid-intestine (**C**,**c**): higher magnification).

**Figure 16:**
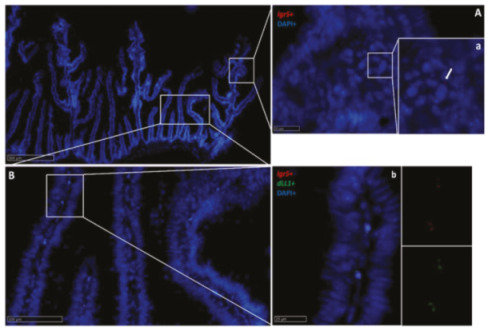
In situ hybridization of *lgr5* and *dll1* mRNA in the second segment of the rainbow trout mid-intestine. *Lgr5* (red dots) expression was low at the complex fold apex (**A**,**a**): higher magnification, arrow) and more intense at the base of the complex folds and in the rest of the second segment of the mid-intestine (**B**,**b**): higher magnification). The two genes were expressed in a few cells found along the villus connective axis (**b**).

Slender, elongated *dll1*^+^ cells were found within the fold epithelium of all intestinal tracts but were more numerous in the pyloric caeca and in the basal part of the complex folds of the second segment of the mid-intestine (Figure 15). *Dll1^+^* cells were also found in the lamina propria along the intestinal tract. Interestingly, epithelial *dll1*^+^ cells were located close to stromal *lgr5*^+^ cells, and stromal *dll1*^+^ cells also expressed *lgr5* (Figure 16).

#### 2.2.6. Wnt3a

**Figure 17:**
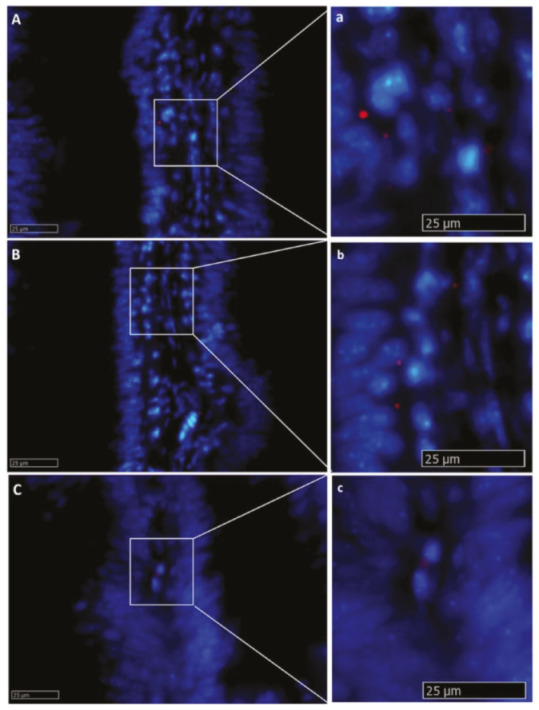
In situ hybridization of *wnt3a* mRNA in the rainbow trout mid-intestine (red dots). The signal was lower in the first segment of the mid-intestine (**A**,**a**): higher magnification), in the pyloric caeca (**B**,**b**): higher magnification), and in the apical part of the complex folds of the second segment of the mid-intestine, compared to the basal part of the complex folds (**C**,**c**): higher magnification) and to the rest of the second segment of the mid-intestine.

**Figure 18:**
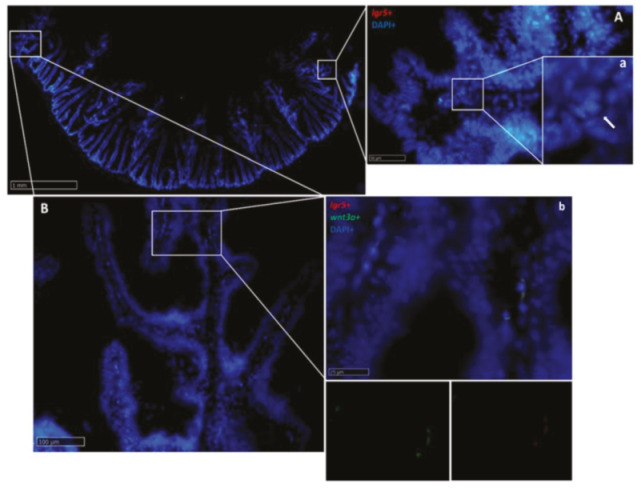
In situ hybridization of *lgr5* and *wnt3a* mRNA in the second segment of the rainbow trout mid-intestine. *Lgr5* (red dots) expression was low at the complex fold apex (**A**,**a**): higher magnification, arrow), but became higher at the base of the complex fold and in the rest of the second segment of the mid -intestine (**B**,**b**): higher magnification), where it colocalized with *wnt3a* along the villus connective axis (**b**).

*Wnt3a* was found in stromal cells along the fold (Figure 17) and colocalized with *lgr5* (Figure 18) and *notch1*. Once again, the expression was higher in the second segment of the mid-intestine than in the first segment of the mid-intestine. Pyloric caeca presented an intermediate intensity of expression.

**Figure 19:**
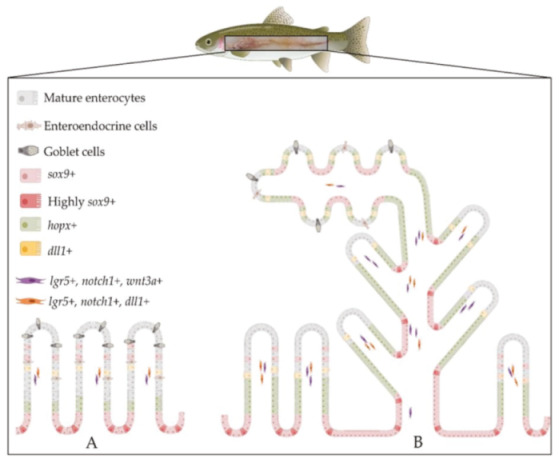
Schematic illustration of the architecture and the organization of the intestinal stem-cell niche in rainbow trout in the first (**A**) and second (**B**) segment of the mid-intestine. *Sox9* was expressed at the base of the fold and the signal was downregulated along the fold length, where *hopx* expression increased and then faded in the upper portion of the folds. *Lgr5* was exclusively expressed in scattered cells located in the lamina propria, where it colocalized with *notch1* and *wnt3a*. *Dll1* also colocalized with *lgr5* in the lamina propria and it was expressed in the epithelium near *lgr5*^+^ stromal cells. The distribution of these markers along the rainbow trout intestine was not homogeneous; rather, we observed the highest expression in the basal part of the complex folds of the second segment of the mid-intestine compared to other districts. Illustrations created with BioRender.com.

The locations of the different molecules expressed in RT intestinal stem cells and in their niche are summarized in Figure 19.

## 3. Discussion

The first aim of this study was to investigate whether the complex nonlinear 3D distribution pattern that we previously described along the RT intestine [5] reflects a similar distribution of functional activities. 

Our preliminary morphological observations were fully coherent with those that we described previously despite, in the first case, animals being raised in standardized indoor conditions, whereas, in this experiment, animals were farmed in open natural-like conditions. This suggests that these morphological features are typical of this species and are not the result of intensive farming or of a specific diet. We did not observe any fold branching in the first segment of the mid-intestine, as described in previous works [5,26], supporting the hypothesis that this phenomenon is not directly related to growth per se, as described by other authors [9], but rather a consequence of stressful conditions [27]. Indeed, in our previous study, fold branching occurred in correspondence with the increase of lipid concentration in the diet and with qualitative changes in mucin composition [5], further supporting this thesis. All this, together with the fact that we did not observe inflammation features [26] such as supranuclear vacuolization of the enterocytes of the first segment of the mid-intestine, nuclear position disparity, or the presence of intraepithelial lymphocytes, confirmed that the all animals were in perfect health and support the physiological significance of our findings.

Peptide transporter 1 (*PepT1*), is a high-capacity, low-affinity transporter and is the main carrier responsible for the uptake of dietary peptides in mammals and fish [28]. *PepT1* protein was localized along the brush border of the absorptive epithelial cells lining the mucosa folds, as previously described in rainbow trout alevins [29]. The signal was highest in the pyloric caeca, while it was moderate in the first segment of the mid-intestine and in the apical part of the complex folds of the second segment of the mid-intestine; however, it was completely absent at their basal part and in the rest of the second segment of the mid-intestine. This is consistent and explains the steady decrease in *PepT1* messenger RNA (mRNA) previously described in salmon [30], rainbow trout [31], and sea bass [32]. Our results are also in agreement with previous observations showing a significant decrease in SGLT-1 in the second segment of the mid-intestine compared to the first portion and to the pyloric caeca, supporting the thesis that the rainbow trout displays three kinetic glucose absorption systems corresponding to the three different intestinal tracts [33,34]. Moreover, the heterogeneous distribution of Fabp2 along the whole intestine that we observed is fully in agreement with a previous study conducted in salmon in which the authors investigated Fabp2 expression and localization along the different tracts [2].

Overall, the correspondence between the complex nonlinear 3D distribution pattern that we described along the RT intestine [5] is reflected only partially by a similar distribution of functional activities. This holds true only in the first segment of the mid-intestine and in the apical part of the complex folds on one side, which are always different from the rest of the second segment of the mid-intestine on the other. This further confirms the heterogeneous nature of the second segment of the mid-intestine mucosa, where the apical part of the complex folds has the same morphology and, presumably, extends the functions of the first segment. Such structure corresponds well to the nutritional needs of the RT short intestine, apt for processing a highly digestible, nutrient-dense diet, high in protein and low in carbohydrate [35], extending the active nutrient transport area typical of the first segment of the mid-intestine [36] in the more distal region. 

The second aim of our work was to determine the mechanisms sustaining the higher proliferation rate that we previously observed in the pyloric caeca and in the second segment of the mid-intestine compared to the other sections. To this purpose, we characterized RT intestinal stem cells and their niche. 

As in mammals, the RT intestinal epithelium undergoes a constant renewal even though it occurs at a slower pace [37]. The renewal mechanisms are driven by an intestinal stem-cell population housed in a defined niche [13]; however, in fish, both the cells and the niche are poorly characterized. In mammals, intestinal stem cells are located in the crypts [11,37], while, in fish, they are found at the fold base [8,38]. To identify RT intestinal stem cells, we used LGR5, the specific marker for rapidly dividing intestinal stem cells identified in mouse, where it is expressed in specific cells at the bottom of the crypts [10,12,13,39]. Medaka is the only other fish species where *lgr5* expression has been investigated, and its mRNA is confined to the base of the intestinal folds [8]. On the contrary, we found *lgr5*^+^ cells exclusively within the lamina propria at the middle of the fold height. This suggests that *lgr5* is unlikely to be a specific marker of RT intestinal stem cells. However, *lgr5* expression in a stromal cell subpopulation housed along the villi axis was also recently described in mouse. These were identified as telocytes, and the ablation of these peculiar cells caused a perturbation of the gene expression pattern of enterocytes, suggesting that their function is to ensure the maintenance of an efficient epithelium at the villus tip [40]. Telocytes have been identified as a subepithelial source of proliferative signals to the stem/progenitor cell compartment of the intestinal stem-cell niche in both mouse [41,42] and human [43,44]. Therefore, it is reasonable to assume that a similar function may also be played by these cells in RT.

While we did not observe *lgr5*^+^ cells at the base of RT intestinal folds, in this location, epithelial cells were expressing different levels of *sox9* along the whole intestine length. This is consistent with *sox9* expression within the mammalian crypts and in the basal portion of medaka [8] and zebrafish [23] intestinal folds. Mouse ISCs not only express *lgr5* but also show a peculiar morphology for which they are named crypt base columnar cells (CBCs) [12]. Interestingly, we observed elongated cells intensely expressing *sox9* in the middle of the intestinal folds base, displaying the same typical position and shape of mouse CBC cells. In mouse intestine, CBC cells are interposed between other *sox9*^+^ cells; similarly, in RT, columnar strongly *sox9*^+^ cells were located among other cells expressing a lower *sox9* signal. Moreover, *sox9*^+^ cells were much more abundant in the second segment of the mid-intestine and the basal part of the complex folds and in the pyloric caeca compared to the first segment of the mid-intestine and the apical part of the complex folds, consistent with the pattern of proliferating cell nuclear antigen (Pcna) expression previously described in this species [5]. Notably, *sox9*^+^ cells did not coexpress other stem-cell markers and they sharply disappeared outside the intestinal fold base, where *sox9* expression was substituted with that of *hopx*. These observations support the hypothesis that this distinctive cell population represents the RT ISCs. 

We also observed scattered, elongated *sox9*^+^ cells along the fold’s axis; therefore, according to our previous observations, they should not express Pcna and, thus, constitute a different subgroup. This is consistent with the identification of two SOX9^+^ cell populations in mouse, one located at the classical crypt base and the other located along the villus epithelium [45]. The latter lacked the typical proliferating markers and simultaneously expressed chromogranin A, the specific marker of enteroendocrine cells. These findings suggested that. in mouse. SOX9 expression along the villus epithelium might detect terminally differentiated secretory cell type. Even if we were not able to verify the expression of chromogranin A in our samples, due to the lack of a reactive specific antibody, this hypothesis may be true in RT as well, because *sox9^+^* cells displayed the same elongated morphology of enteroendocrine cells described in zebrafish [46]. 

In mouse intestine, HOPX^+^ cells are rare and restricted to the +4 position of the intestinal crypt. Conversely, in RT, *hopx*^+^ cells were abundant, absent at the fold base, but detected in the epithelial cells along the fold. Few *hopx*^+^ cells were also found within the fold connective axis. Taken together, these discrepancies lead us to believe that *hopx*^+^ cells in RT cannot be considered the functional equivalent of quiescent ISC as in the mammal intestine. Since the *hopx*^+^ cell location pattern also matches Pcna expression, these cells are more likely to correspond to the transient amplifying population, an undifferentiated population in transition toward differentiation. 

Consistently with the *sox9* expression pattern, *hopx*^+^ cells were more abundant in the pyloric caeca and even higher in the second segment of the mid-intestine and in the basal part of the complex folds, while a smaller population was detected in the first segment of the mid-intestine and in the apical part of the complex folds of the second segment of the mid-intestine. Interestingly, in piglet intestine, *hopx*^+^ cells also did not follow a linear distribution along the whole intestine and were more numerous in the colon [11].

NOTCH1 is another mouse ISC marker that is expressed in the crypt base, specifically by LGR5^+^ cells, inducing their differentiation toward the absorptive lineages. In RT, *notch1*^+^ cells were scattered along the fold connective axis, while it was also expressed by the same cells expressing *lgr5*. The combined expression of these two molecules must have a different function in this context, and further research is needed to clarify this aspect. 

Close to *lgr5*^+^/*notch1*^+^ stromal cells, elongated *dll1*^+^ epithelial cells were found in RT intestine. They were located along the fold length, and their proximity suggested an interaction between these two cell types. This is consistent with the expression of DeltaD, the *dll1* homolog in the zebrafish intestinal epithelium [22]. In mouse small intestine, Dll1 is expressed by Paneth cells at the crypt base [19]. Despite their fundamental role, the presence of bona fide Paneth cells has not been detected in many mammals and fish species including RT [5,11]. However, DLL1 is also expressed in mouse colon, where Paneth cells are absent [47]. Taken together, these observations suggest the hypothesis that, in those species where typical Paneth cells are not present, another cell type may functionally substitute them, actively interacting with ISCs. 

Few *dll1*^+^ cells were also found within the stromal axis of the folds, but their functional role and their implications in the intestinal stem-cell niche are unclear. In zebrafish, in the absence of the Delta–Notch signaling pathway, secretory differentiation becomes the default, and the lateral inhibition of a functional Delta–Notch signaling restores a balanced mixture of absorptive and secretory cells [22]. In the mouse intestine, Paneth cell maturation and differentiation are driven and promoted by Wnt signaling, the evolutionarily conserved pathway that contributes to stem-cell maintenance. Recent studies suggested a redundancy of Wnt sources; indeed, it is expressed by stromal and epithelial cells [48,49]. In RT, similarly to mouse small intestine, *wnt3a* is expressed by a subepithelial cell population. However, while, in mouse, WNT3A^+^ cells are in the pericryptal region, in RT, *wnt3a*^+^ cells were scattered along the fold connective axis. 

Recent studies of mouse small intestine demonstrated that telocytes also express WNT3A [49]. Interestingly, in RT, the stromal *wnt3a*^+^ cell population colocalizes with that expressing *lgr5*. This expression pattern is consistent with that of mouse telocytes, supporting the hypothesis that telocytes are also active in the RT intestinal mucosa. 

Therefore, our results indicated that mammal intestinal stem-cell markers are also expressed in the RT intestine; however, their localization, the niche architecture, and the interactions among these markers are not conserved. In fact, the trout functional equivalent of *lgr5* seems to be *sox9*, since *sox9*^+^ cells showed the peculiar location and shape of CBCs in mouse intestine. On the other hand, while, in mammals, LGR5 is the specific crypt epithelial stem-cell marker, in trout, it is exclusively expressed by a stromal cell population along the fold. These cells could be telocytes, a specialized mesenchymal cell type that was recently indicated as a fundamental component of the intestinal stem-cell niche, able to support and regulate the epithelium interacting with nearby *dll1*^+^ cells. 

The absence of typical Paneth cells and the presence of a specific epithelial cell population displaying an expression pattern similar to mouse Paneth cells suggests that this specialized cell type might be considered its functional equivalent.

Lastly, our data indicated that the high renewal rate displayed by pyloric caeca and by most of the second segment of the mid-intestine is fueled by a more extensive stem-cell population.

## 4. Materials and Methods 

### 4.1. Sample Collection and Processing

A total of five adult rainbow trout (RT), four females and one male, weighing approximately 500 g, with a length between 31 and 33 cm, were collected during the month of April from fish culture ponds at the Laghi Verdi s.n.c. trout farm (Como, Italy). Individuals were euthanized according to Annex IV European Union (EU) guideline 2010/63, during nonexperimental clinical veterinary practices. 

The sample numerosity was in the same range (3–6) of previous qualitative descriptive studies of fish intestinal anatomy [8,9,38].

**Figure 20:**
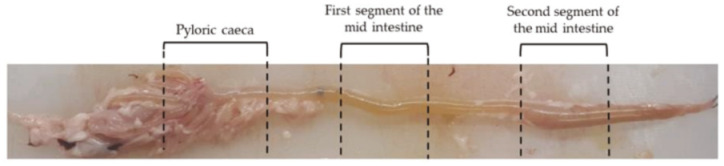
Regions of the rainbow trout mid-intestine where samples were collected: pyloric caeca, first segment of the mid-intestine, and second segment of the mid-intestine.

A longitudinal incision along the ventral line was performed and the entire gastrointestinal (GI) tract was removed. Samples of the pyloric caeca, as well as of the first and second segment of the mid-intestine (Figure 20), were collected and promptly fixed in 10% neutral buffered formalin for 24 h at room temperature, subsequently dehydrated in graded alcohols, cleared with xylene, and embedded in paraffin.

### 4.2. Histology and Immunohistochemistry

Thin sections of 4 μm were stained with hematoxylin and eosin (H&E) to evaluate sample morphology. Subsequently, other sections were used for immunostaining purposes. Peptide transporter 1 (Pept-1) and sodium–glucose co-transporter-1 (Sglt-1) were characterized through immunohistochemistry using the Avidin–Biotin Complex method (VECTASTAIN^®^ Elite^®^ ABC, Vector Laboratories, Burlingame, CA, USA) following the manufacturer’s indications. In brief, sections were immersed in 10 mM sodium citrate buffer, 0.05% Tween-20 (pH 6) and brought to boiling; then, endogenous peroxidase was quenched, applying it to slides with 3% H_2_O_2_ in methanol solution for 15 min. Nonspecific binding was prevented by incubating sections in Normal Blocking Serum (Vectastain ABC Elite KIT, Burlingame, CA, USA) for 30 min at room temperature. Then, samples were incubated with anti-PEPT1 mouse monoclonal antibody (Santa Cruz Biotechnology, sc-373742, Heidelberg, Germany) 1:100 or with anti-SGLT-1 rabbit polyclonal antibody (Millipore Corporation, 07-1417, Darmstadt, Germany) 1:10,000 diluted in 4% bovine serum albumin (BSA) in phosphate-buffered saline (PBS) with 0.05% Tween-20 for 60 min at room temperature in a humidified chamber. Antibody specificity was previously validated in rainbow trout intestine [29,33]. Sections were then incubated with appropriate biotinylated secondary antibody followed by the avidin–biotinylated horseradish peroxidase (HRP) complex (Vectastain ABC Elite KIT, Burlingame, CA, USA) for 30 min at room temperature each. Signal development was performed incubating slides with ImmPACT 3,3′-diaminobenzidine DAB substrate (Vector Laboratories, SK-4105, Burlingame, CA, USA). Sections were then briefly counterstained with hematoxylin solution modified according to Gill I, dehydrated, and permanently mounted with a synthetic mounting medium (Histo-Line laboratories, R0081, Milano, Italy). 

Fatty-acid-binding protein 2 (Fabp2) was localized through indirect immunofluorescence. Briefly, thin sections were deparaffinized, rehydrated, and brought to boiling in 10 mM sodium citrate buffer, 0.05% Tween-20 (pH 6) for antigen retrieval. Nonspecific binding was prevented by incubating slides in 10% donkey serum in PBS for 30 min at room temperature. Then, samples were incubated with anti-FABP2 goat polyclonal antibody (Novus Biologicals, NB100-59746 Littleton, CO, USA) 1:150 diluted in 4% BSA in PBS for 60 min at room temperature in a humidified chamber. Primary antibody specificity was previously validated [2]. Subsequently, slides were incubated with secondary antibody Alexa Fluor^TM^ 594 donkey anti-goat (Life Technologies Corporation, A11058 Willow Creek Road, OG, USA) 1:1000 diluted in PBS for 30 min at room temperature. Sections were then were counterstained with 4′,6-diamidino-2-phenylindole (DAPI) and mounted with ProLong™ Gold Antifade Mountant (ThermoFisher Scientific, Waltham, MA, USA). Secondary antibody controls were performed by omitting the primary antibody.

### 4.3. Target Probe Design and Fluoresce in Situ Hybridization (FISH)

Since mouse is the species in which intestinal stem cells have been studied most, we selected the following mouse intestinal stem-cell markers as target genes: SRY-box 9 (*Sox-9*), leucine-rich repeat-containing G-protein-coupled receptor 5 (*Lgr5*), homeodomain-only protein (*Hopx*), Notch receptor 1 (*Notch1*), Delta-like protein 1 (*Dll1*), and Wnt family member 3A (*Wnt3a*). Expression of these genes in rainbow trout intestine was confirmed by PCR, and the amplicon sequences were sent to Advanced Cell Diagnostics (ACD) for the design of custom in situ hybridization probes. Fluorescent in situ hybridization was performed using Multiplex Fluorescent Reagent Kit V2 (RNAscope technology, Advanced Cell Diagnostics, San Francisco, CA, USA) according to the manufacturer’s instructions. This assay allows simultaneously visualizing two targets when specific customized probes are conjugated with different channels. Briefly, thin sections of 5 µm were first heated on a stove and immersed in xylene to encourage paraffin removal. Samples were later incubated with hydrogen peroxide (Advanced Cell Diagnostics, San Francisco, CA, USA) and brought to boiling in a target retrieval solution (Advanced Cell Diagnostics, San Francisco, CA, USA). Subsequently, slides were exposed to Protease plus (Advanced Cell Diagnostics, San Francisco, CA, USA) to allow probes to reach their defined target. Afterward, sections were incubated with specific probes diluted 1:50 in diluent buffer in a HybEZ oven (Advanced Cell Diagnostics, San Francisco, CA, USA) for 2 h at 40 °C. Probes were conjugated with different specific channels in order to allow multiplex comparison (Table 1).

Signal amplification was performed by incubating samples in signal amplification solutions 1, 2, and 3. The signal was the developed incubating slides with the appropriate fluorophore (OPAL 520 or OPAL 570, Akoya biosciences, Marlborough, MA, USA) diluted 1:750 in tyramide signal amplification (TSA) buffer. Sections were then counterstained with DAPI and mounted with ProLong™ Gold Antifade Mountant (ThermoFisher Scientific, Waltham, MA, USA). The mRNA quality and integrity were checked using a constitutive control gene (PPiB—Peptidylprolyl isomerase B), while negative controls were performed by incubating slides with a probe specific for the *Bacillus subtilis* dihydrodipicolinate reductase (dapB) gene. 

According to the ACD RNAscope^®^ indications, the signal from a single mRNA molecule is detected as a punctate dot, whereas larger dots (cluster) result from many mRNA molecules. Furthermore, high RNA expression can be easily seen at 10× magnification, whereas low RNA expression typically requires a 40× magnification examination. 

Images were acquired using a NanoZoomer S60 Digital slide scanner (Hamamatsu photonics, Hamamatsu city, Japan) and were collected at low and high magnification to show general tissue distribution along the folds and within enterocytes, respectively.

## 5. Conclusions

We previously described the RT mid-intestine peculiar 3D structure and, here, we presented a detailed qualitative description indicating that the apical portion of the complex folds present in the second segment of the mid-intestine provides a morphological and functional extension to the first segment. The remaining portion of the intestine, the pyloric caeca, and the rest of the second segment of the mid-intestine do not share the same brush border enzyme expression pattern but share the same epithelial morphology and the same elevated renewal rate. Although, at present, we have no data that may explain why these two portions undergo a higher wear and tear than the rest, we characterized the RT ISC morphological and molecular architecture. Evidence indicates that, while the molecular repertoire is conserved compared to mammals, their 3D distribution is not, and it also partially diverges from that of other fish, confirming the extreme variety that characterizes this vast group.

Even if no histological and molecular details are known, a similar complex nonlinear 3D pattern of the mid-intestine was previously described also in the Atlantic salmon; therefore, our results are likely applicable to salmonids, a group of species very valuable for the aquaculture industry. Our data provide the qualitative parameters that describe the organization of their intestinal stem-cell niche, adding to the array of innovative observations that are emerging from recent morpho functional studies [50]. 

In the future, quantitative measurements of these parameters will enable understanding the impact that different innovative feeds may have on the gut health of these species. To measure the response of the ISC niche to different degrees of nutritional stress will provide functional clues, currently missing but greatly needed, to fine-tune our tools for improving aquaculture sustainability.

## Figures and Tables

**Table 1:** Probe targets and corresponding conjugated channels.

mRNA Target	Channel	Cat.N.
*lgr5*	Channel 1	847731
*hopx*	Channel 2	847761-C2
*wnt3a*	Channel 2	847771-C2
*sox9*	Channel 3	847751-C3
*notch*	Channel 3	847741-C3
*dll1*	Channel 3	853451-C3

## Abbreviations

RT	Rainbow trout
PEPT1	Peptide transporter 1
SGLT1	Sodium–glucose/galactose transporter 1
FABP2	Fatty-acid-binding protein 2
ISCs	Intestinal stem cells
LGR5	Leucine-rich-repeat-containing G-protein-coupled receptor 5
HOPX	Homeodomain-only protein
SOX9	SRY-box 9
NOTCH1	Notch receptor 1
DLL1	Delta-like protein 1
WNT3A	Wnt family member 3A
CBCs	Crypt base columnar cells
GI	Gastrointestinal
HRP	Horseradish peroxidase
DAB	3,3′-Diaminobenzidine
DAPIFISH	4′,6-diamidino-2-phenylindoleFluorescence in situ hybridization
